# Prevalence of Depression and Coping Strategies Among Head and Neck Cancer Patients: A Hospital-Based Study

**DOI:** 10.7759/cureus.43687

**Published:** 2023-08-18

**Authors:** Pradeep TS, Nagireddy Reddy Sreenath, Vamsi Krishna

**Affiliations:** 1 Community Medicine, Sri Devaraj Urs Medical College (SDUMC) Sri Devaraj Urs Academy of Higher Education and Research (SDUAHER), Kolar, IND; 2 Preventive Medicine, Sri Devaraj Urs Medical College (SDUMC) Sri Devaraj Urs Academy of Higher Education and Research (SDUAHER), Kolar, IND

**Keywords:** diversion, positive focus, mental health, cancer coping, depression, head and neck cancer

## Abstract

Background

The burden of cancer is increasing in all countries and so is in India. Among Indian males, lung, mouth, and tongue are very common cancers. Cancer is a life-threatening stressful condition, and cancer survivors report negative effects which decrease the quality of life because of long-term post-treatment effects. When cancer treatment is ongoing, patients express mixed emotion which is happiness and relief. This study aimed to find out the prevalence of depression and assess the coping strategies among the same head and neck cancer patients.

Material and methods

This cross-sectional study was conducted at the outpatient level in the Department of Radiotherapy, Sri Devaraj Urs Medical College (SDUMC), Sri Devaraj Urs Academy of Higher Education and Research (SDUAHER), Kolar, for a period of three years. The sample size was calculated based on the previous study. Head and Neck cancer patients who are more than 18 years old and histologically diagnosed with cancer were included in the study, and head and neck cancer patients with previously diagnosed mental illness or patients with mental health medications or chronically debilitated cancer were excluded. For sociodemographic details, a pretested semi-structured questionnaire was used. To assess depression Zung Depression Scale was used. To assess coping strategy the Cancer Coping Questionnaire was used. All data collected by interview technique which will last not less than 20 minutes. All data were entered in a Microsoft Excel sheet and analyzed using SPSS version 22 (Armonk, NY: IBM Corp.). To compare between groups, t-test and ANOVA were used with a statistically significant p<0.05.

Results

Of 188 head and neck cancer patients, 117 (62.2%) aged 31-60 years, 136 (72.3%) were females, 121 (64.4%) belonged to rural background, 143 (76.1%) were illiterates, 105 (55.9%) belonged to joint family, and 110 (58.5%) belonged to class IV modified BG Prasad classification. This study showed 16% of head and neck cancer patients had depression according to the Zung Depression Scale. With respect to the coping domain, cancer patients from rural backgrounds had higher coping scores, and illiterate had higher scores. With respect to the positive focus coping subdomain, male cancer patients had higher scores compared, to rural cancer patients, who had higher scores, and illiterate had higher scores. With respect to the diversion domain, rural cancer patients had higher scores, illiterate patients had higher scores, and this difference was statistically significant. With respect to the interpersonal scale domain, rural patients, patients belonging to joint families, patients aged more than 60 years, and illiterate patients had higher scores compared with literates, and these were statistically significant with p<0.05. This study demonstrated that the presence of depression had no impact on coping domains, and there was no significant correlation between depression and coping scores.

Conclusion

Mental health must be thoughtfully considered among cancer patients as cancer with depression may have a negative impact on their experience with cancer. Cancer keeps growing as a public health problem and all cancer-treating hospitals should work on various preventable measures for reducing the future burden of various health dimensions affected by cancer. The very intention of treating cancer should be improving the survival of the diagnosed case and this needs targeted mental health intervention.

## Introduction

The burden of cancer, which is typically life-threatening, is increasing steadily throughout the world. However, a diagnosis of cancer can lead to serious psychological problems. Cancer is often considered synonymous with hopelessness, unbearable pain, fear, and certain death among cancer patients [1]. Every year, more than a thousand new cases per 1,00,000 population of all types of cancers are detected in India, which is undeniably a major public health concern [2]. Although there are well-established treatment options for most diagnosed cancers, as well as impressive research in new drug discoveries, cancer survivors report decreased quality of life because of long-term post-treatment side effects and ongoing physical, psychological, and social problems caused by cancer. When cancer treatment is ongoing, patients often face mixed emotions, including happiness and relief that come at the end of the treatment, but also fear and anxiety of relapse. Therefore, it is undeniable that cancer is a traumatic stressor [3].

Due to increased early detection and advances in cancer treatments, more cancer patients survive for longer, yet often with one or more disabilities. Although various factors of the economic burden among different cancers have been mitigated by the existing public health system in India, patients still incur some out-of-pocket costs, adding to the financial stress of the cancer patient, which can also affect mental health [4]. Among all cancer types, head and neck cancers (HNCs) pose a significant healthcare burden in India, as they are very common due to various reasons, such as tobacco consumption in smokeless forms and ignorance of the complications of the same. Unfortunately, HNC patients often present in an advanced stage of the disease due to delayed diagnosis. In addition, when these patients are diagnosed with advanced cancer at the oncology clinic, the focus of care is usually on developing a treatment plan for cancer rather than other dimensions of health. Such patients often experience spiritual and emotional distress along with frightening physical symptoms [5]. Late diagnosis can lead to long-term health consequences and a lower HNC survival rate, impacting the physical, emotional, and psychological needs of cancer patients [6].

Coping is defined broadly as an effort used to minimize distress associated with negative life experiences. Coping strategies are patients’ ability to face the reality of their life situation and to develop more adaptive coping responses. Stressful experiences, such as cancer diagnosis, have long been posited as a contributor to cognitive changes [7,8]. Adaptive coping strategies allow patients to spend the rest of life with a positive focus on their poor health.

Overall, maladaptive coping in cancer patients could be the result of difficulty in establishing diagnostic criteria, as neuro-vegetative signs and symptoms may be attributable either to depression or physical illness [9]. There is also growing interest in understanding psychosocial concerns, depression, and coping strategies among cancer patients. Cancer patients often find it hard to cope with the psychological consequences of cancer and deal with existential issues, such as fear of death, isolation, rejection, meaninglessness, questioning the meaning of life, and threats to self-identity, very few patients do not experience many problems in dealing with the aftermath of their disease. All these factors can be powerful predictors of mental health illness among cancer patients [10].

A diagnosis of HNC can be devastating, and deciding on the appropriate treatment can be complicated and daunting. Similar to various other cancers, patients with HNC may suffer from variable degrees of functional impairment that are related to speaking, swallowing, breathing, taste, and smell, as well as facial disfigurement during the courses of illness and treatment. They are also at higher risk of having emotional distress than any other form of cancer amid the loss of these functions, pushing toward mental illness and poor coping abilities. Therefore, we performed this study to determine the prevalence of depression among HNC patients, assess the coping strategies among HNC patients, and investigate the correlation between depression and coping scores among HNC patients.

## Materials and methods

We carried out a cross-sectional study at the outpatient level in the Department of Head and Neck Oncology, Sri Devraj Urs Medical College, Kolar, for a period of three years from 2019 to 2022. We calculated the sample size based on a previous study. As the prevalence of depression among HNC patients was 18.5%, under a relative error of 6%, the estimated sample size was 165 [11]. We calculated the sample size using OpenEpi software. On the same HNC patients, coping strategies were assessed. The participants who were more than 18 years, diagnosed with histologically confirmed HNC at any stage irrespective of their surgical status, and able to communicate were included in this study. Those with previously diagnosed mental illness, those on any mental health medications, those who had received any psychological counseling, and those who are chronically bedridden or unable to communicate with the interview were excluded from the study. We used a pre-tested semi-structured questionnaire to assess the sociodemographic characteristics of the participants.

We used Zung Self-Rating Depression Scale to assess depression in the HNC patients which is freely available online. The scale is an established norm-referenced screening measure used to identify the presence of depressive disorders in adults, wherein a short survey is used to quantify the depressed status of a patient. There are 20 items on the scale, which assess the following four common characteristics of depression: the pervasive effect, the physiological equivalents, other disturbances, and psychomotor activities. There are 10 positively worded and 10 negatively worded questions. Each question is scored on a scale of 1-4, i.e., a little of the time, some of the time, a good part of the time, and most of the time, respectively. Scores are classified as follows: normal (25-49, mildly depressed (50-59), moderately depressed (60-69), and severely depressed (≥70) [12].

We used the Cancer Coping Questionnaire (CCQ) to assess coping strategies among the HNC patients. The 21-item questionnaire uses a brief, self-rating scale designed to measure coping strategies (i.e., effective and ineffective ways to cope with a stressful life event). The scale is usually used in counseling settings to interpret the helpful and unhelpful patterns someone responds to stressors. The questionnaire comprises the Total Individual Scale (items 1-14) and the following subscales: coping (items 2, 6, 7, 11, 12), positive focus (items 1, 9, 14), diversion (items 3, 4, 8), planning (items 5, 10, 13), and interpersonal (items 15-21). A high CCQ score indicates better physical or cognitive efforts to disengage from the stressor (i.e., better coping) [13,14].

We obtained written informed consent from each HNC patient after informing them about the benefits and risks involved in the study. We made every effort to maintain the confidentiality of the study participants. Participation in the study was completely voluntary. We also maintained the confidentiality of the participants by not recording their names or personal details. We started the study after receiving approval from our Institutional Ethics Committee (#DMC/KLR/IEC/190/2023-24). We collected all data via the interview technique, which did not last more than 20 minutes for each patient. We entered all data into a Microsoft Excel spreadsheet and analyzed them using SPSS version 22 (Armonk, NY: IBM Corp.). Student’s t-test and one-way analysis of variance were used for comparisons between groups, with p<0.05 considered statistically significant. Logistic regression analysis was also done.

## Results

Out of 188 cancer patients, 117 (62.2%) belonged to 31-60 years, 136 (72.3%) were females, 121 (64.4%) belonged to rural background, 126 (67%) did not work for wages, 143 (76.1%) were illiterates, 105 (55.9%) belonged to joint family, and 110 (58.5%) belonged to class IV modified BG Prasad classification (Table 1). Out of 188 cancer patients, 30 (16%) had depression according to the Zung Depression Scale. Out of 30 head and neck cancer patients, 22 (73.3%) had moderate depression, and eight (26.7) had severe depression.

**Table 1:** Distribution of cancer patients according to clinico-sociodemographic profile.

Variables		Frequency	Percent
Age (years)	30-60	117	62.2
	>60	71	37.8
Gender	Male	52	27.7
	Female	136	72.3
Place of living	Urban	67	35.6
	Rural	121	64.4
Occupation	Do not work	126	67.0
	Work for wages	62	33.0
Education	Illiterate	143	76.1
	Literate	45	23.9
Type of family	Nuclear	83	44.1
	Joint family	105	55.9
Treatment	Intravenous chemotherapy	12	6.4
	Oral chemotherapy	176	93.6
Duration of treatment	<6 months	49	26.1
	6-12 months	85	45.2
	>12 months	54	28.7
Socioeconomic status according to modified BG Prasad classification 2022	II	15	8.0
	III	63	33.5
	IV	110	58.5

Cancer patients with rural backgrounds had better CCQ scores suggesting better coping compared with urban patients, illiterate patients had better CCQ scores suggesting better coping compared with literate patients, and duration of treatment less than six months had better CCQ scores compared with those more than six months. All these differences were statistically significant with a p-value less than 0.05 (Table 2).

**Table 2:** Comparison of Cancer Coping Questionnaire (CCQ) scores with various factors.

Clinico-sociodemographic factors		Mean±SD	p-Value
Age (years)	30-60	61.6±6.9	0.4
	>60	63.0±7.3	
Gender	Male	63.0±6.9	0.3
	Female	61.8±7.1	
Place of living	Urban	60.4±5.5	0.01
	Rural	63.0±7.7	
Type of family	Nuclear	61.2±6.5	0.18
	Joint	62.8±7.5	
Education status	Illiterate	62.7±6.9	0.04
	Literate	60.2±7.4	
Occupation	Does not work	62.0±6.4	0.6
	work for wages	62.4±8.3	
Treatment	Intravenous chemotherapy	64.4±9.7	0.25
	Oral chemotherapy	62.0±6.9	
Duration of treatment	<6 months	63.9±6.4	0.01
	6-12 months	61.8±7.2	
	>12 months	61.0±7.4	
Modified BG Prasad classification 2022	II	63.8±8.7	0.5
	III	62.4±8.0	
	IV	61.7±6.3	

With respect to the coping domain, cancer patients from rural backgrounds had higher coping scores (14.8±2.1) compared with urban patients (13.6±1.5), illiterate had higher scores (14.6±1.9) compared with literate cancer patients (13.6±2.1), and this difference was statistically significant. With respect to the positive focus domain, male cancer patients had higher scores (9.4±1.3) compared with females (8.7±1.4), rural cancer patients had higher scores (9.1±1.4) compared with urban (8.6±1.4), and illiterates (9.7±1.3) had higher scores compared with literates (8.2±1.4), and this difference was statistically significant. With respect to the diversion domain, rural cancer patients had higher scores (9.1±1.4) compared with urban (8.6±1.4), illiterate patients had higher scores (8.7±1.3) compared with literate (8.2±1.4), and this difference was statistically significant. With respect to the interpersonal scale domain, rural patients (21.3±2.9), patients belonging to joint families (21.4±2.5), patients aged more than 60 years (21.6±2.5), and illiterate patients (21.3±2.9) had higher scores with statistical significance (Table 3).

**Table 3:** Comparison of various factors with coping domains.

Clinico-sociodemographic factors		Coping	Positive focus	Diversion	Planning	Interpersonal scale
Gender	Male	14.2±2.2	9.4±1.3	8.6±1.4	9.0±1.6	21.6±3.0
	Female	14.5±1.9	8.7±1.4	8.5±1.3	8.9±1.5	21.0±2.5
	p-value	0.3	0.002	0.9	0.4	0.6
Place of living	Urban	13.6±1.5	8.6±1.4	8.6±1.4	8.5±1.2	20.9±2.2
	Rural	14.8±2.1	9.1±1.4	9.1±1.4	9.1±1.6	21.3±2.9
	p-value	0.001	0.02	0.02	0.01	0.02
Type of family	Nuclear	14.2±1.8	8.9±1.3	8.4±1.2	9.0±1.4	20.8±2.9
	Joint	14.5±2.2	8.9±1.4	8.7±1.4	8.5±1.6	21.4±2.5
	p-value	0.2	0.8	0.4	0.4	0.02
Age in years	30-60 years	14.4±2.0	8.9±1.4	8.5±1.4	9.2±1.6	20.9±2.7
	>60 years	14.4±2.0	8.9±1.5	8.7±1.3	8.7±1.4	21.6±2.5
	p-value	0.7	0.7	0.9	0.4	0.02
Education status	Illiterate	14.6±1.9	9.7±1.3	8.7±1.3	8.7±1.4	21.3±2.4
	Literate	13.6±2.1	8.2±1.4	8.2±1.4	9.1±1.5	19.8±3.3
	p-value	0.02	0.03	0.03	0.04	0.02
Occupation	Doesn’t work	12.4±1.8	8.8±1.4	8.5±1.3	8.9±1.4	21.0±2.3
	Work for wages	14.3±2.4	9.1±1.4	8.6±1.5	8.9±1.7	21.4±3.2
	p-value	0.03	0.66	0.9	0.8	0.8
Treatment	Intravenous chemotherapy	15.4±2.6	9.4±1.7	8.8±1.9	9.2±2.0	21.5±3.3
	Oral chemotherapy	14.3±1.9	8.9±1.4	8.5±1.3	8.9±1.5	21.1±2.6
	p-value	0.08	0.7	0.6	0.6	0.4
Modified BG Prasad classification 2022	II	63.8±8.7	9.2±1.3	9.0±1.5	9.0±1.5	21.0±3.7
	III	62.4±8.0	8.7±1.6	8.5±1.6	8.5±1.6	21.4±3.0
	IV	61.7±6.3	9.0±1.3	8.5±1.2	8.5±1.2	21.1±2.3
	p-value	0.4	0.7	0.4	0.4	0.7
Duration of treatment	<6 months	14.5±2.0	9.5±1.1	8.8±1.4	8.8±1.4	21.8±2.8
	6-12 months	14.4±2.1	8.9±1.5	8.5±1.4	8.5±1.4	21.0±2.5
	>12 months	14.4±2.1	8.4±1.3	8.4±1.3	8.4±1.3	20.9±2.6
	p-value	0.7	0.001	0.4	0.4	0.4

Table 4 shows that 18.3% of those who had depression were aged more than 60 years compared with 30-60 years and this association between age and depression was not statistically significant; 16.9% of those who had depression were females compared with males and this association between gender and depression was not statistically significant. Further, 16.5% of those who had depression were from rural backgrounds compared with urban backgrounds, and this association between place of living and depression was not statistically significant; 16.1% of those who had depression did not work for wages compared with those who work for wages and this association between depression and occupation was not statistically significant, 16.2% of those who had depression belonged to a joint family compared with a nuclear family and this association between type of family and depression was not statistically significant. Also, 16.1% of those who had depression were illiterates compared to literates and this association between educational status and depression was not statistically significant; 20% of those who had depression were taking treatment for 6-12 months, however, this association between duration of treatment and depression was not statistically significant. Table 4 also shows that 26.7% of those who had depression belonged to the modified BG Prasad classification 2022, however, this association between socioeconomic status and depression was not statistically significant; and 83.3% of those who received intravenous chemotherapy had depression compared with oral chemotherapy and this association between treatments was statistically significant with a p-value less than 0.05.

**Table 4:** Association between depression and various other clinico-sociodemographic characters. P-value less than 0.05 is considered statistically significant.

Clinico-sociodemographic factors		No depression	Depression	p-Value*
Age (years)	30-60	100 (85.5%)	17 (14.5%)	0.312
	>60	58 (81.7%)	13 (18.3%)	
Gender	Male	45 (86.5%)	7 (13.5%)	0.369
	Female	113 (83.1%)	23 (16.9%)	
Place of living	Urban	57 (85.1%)	10 (14.9%)	0.47
	Rural	101 (83.5%)	20 (16.5%)	
Occupation	Doesn’t work	106 (84.1%)	20 (15.9%)	0.56
	Work for wages	52 (83.9%)	10 (16.1%)	
Type of family	Nuclear	70 (84.3%)	13 (15.7%)	0.543
	Joint	88 (83.8%)	17 (16.2%)	
Education	Illiterate	120 (83.9%)	23 (16.1%)	0.38
	Literate	38 (84.4%)	7 (15.6%)	
Treatment	Intravenous chemotherapy	2 (16.7%)	10 (83.3%)	0.001
	Oral chemotherapy	156 (88.6%)	20 (11.4%)	
Duration of treatment	<6 months	45 (91.8%)	4 (8.2%)	0.57
	6-12 months	68 (80.0%)	17 (20.0%)	
	>12 months	45 (83.3%)	9 (16.7%)	
Modified BG Prasad classification 2022	II	11(73.3%)	4 (26.7%)	0.23
	III	56 (88.9%)	7 (11.1%)	
	IV	91 (82.7%)	19 (17.3%)	

HNC patients who do not work for wages had higher odds of 1.16 (0.37-3.61) of having depression, however, it was not statistically significant. Male HNC patients had higher odds of 1.95 (0.6-6.3) of having depression, however, it was not statistically significant. HNC patients who had received intravenous chemotherapy had higher odds of 65.4 (10.9-394.0) of having depression and it was statistically significant (Table 5).

**Table 5:** Binary logistic regression between depression and various other clinico-sociodemographic characters. SE: standard error; B: regression coefficient; HNC: head and neck cancer

Clinico-sociodemographic factors	B	SE	p-Value	Adjusted odds ratio	95% CI for odds ratio	
					Lower	Upper
HNC patients Age less than 30 years	-0.153	0.517	0.767	0.858	0.311	2.364
Illiterates who are HNC patients	-0.055	0.611	0.929	0.947	0.286	3.138
HNC patients who do not work for wages	0.149	0.579	0.796	1.161	0.373	3.613
Receiving intravenous chemotherapy	4.184	0.914	0.000	65.644	10.936	394.041
Duration of treatment less than 6 months	-1.280	0.764	0.094	0.278	0.062	1.243
Duration of treatment 6 months to 12 months	-0.004	0.548	0.994	0.996	0.341	2.912
Modified BG PRASAD class II	-0.004	0.947	0.996	0.996	0.156	6.370
Modified BG PRASAD class III	-1.203	0.668	0.072	0.300	0.081	1.112
Male HNC patients	0.668	0.601	0.266	1.951	0.600	6.342
Urban HNC patients	-0.415	0.529	0.433	0.660	0.234	1.864
Constant	-1.542	0.900	0.087	0.214	-	-

Table 6 shows that having depression and no depression had nothing to contribute to the cancer coping domain suggesting that mental health status like depression was not associated with coping strategies in HNC patients.

**Table 6:** Comparison of various domains with cancer patients having depression and no depression.

Domains of Cancer Coping Questionaire		Mean±SD	p-Value
Interpersonal scale	Normal range	21.2±2.7	0.6
	Depression	21.0±2.5	
CCQ	Normal range	62.0±6.9	0.5
	Depression	62.5±7.8	
Coping	Normal range	14.3±2.0	0.4
	Depression	14.7±2.3	
Positive focus	Normal range	8.9±1.4	0.2
	Depression	9.1±1.3	
Diversion	Normal range	8.5±1.3	0.4
	Depression	8.6±1.5	
Planning	Normal range	8.9±1.5	0.5
	Depression	8.9±1.6	
Overall coping	Normal range	40.8±5.1	0.1
	Depression	41.4±6.1	

## Discussion

Out of the 188 HNC patients, 117 (62.2%) were 31-60 years old, 136 (72.3%) were female, 121 (64.4%) belonged to a rural background, 126 (67%) were unemployed, 143 (76.1%) were illiterate, the majority took oral chemotherapy drugs, 105 (55.9%) belonged to a joint family, and 110 (58.5%) had a class IV modified BG Prasad classification.

Our results showed that 16% of the studied HNC patients had depression according to Zung Self-Rating Depression Scale. Chemotherapy treatment modes had a statistically significant association with depression and regression analysis; the present study showed that HNC patients receiving intravenous chemotherapy had higher odds of having depression. A systematic review conducted by Korsten et al. shows that the prevalence of depression among HNC patients is high because of various factors, such as gender, wherein females are usually more affected than males. Family can play a protective role, as living alone was found to be a risk factor for depression in HNC patients. Income is also a factor, especially in cancer patients from a lower socioeconomic status, who are at high risk for developing depression. Along with that disease stage, chemotherapy, radiotherapy, and other comorbidities can significantly influence depression in HNC patients [15].

A study on HNC patients in the United States showed a depression rate of 23% before cancer treatment, which accounted for decreased radiotherapy compliance for HNC. The study also showed that depression played a significant role in reducing survival, as well as treatment adherence and completion [16]. All these findings were similar to those of the present study. According to a systematic review of 12 articles, changes in weight, sleep disturbances, and loss of appetite are usually present in HNC patients, however, these neuro-vegetative symptoms are likely to be disease- or treatment-related in people diagnosed with cancer and, therefore, not good indicators of depression in this population [17].

Mental health must be thoughtfully considered in cancer patients, depression may have a negative impact on their experience with cancer, treatment adherence, overall feelings of well-being, and adjustment to the stressful event. Lewandowska et al.'s study on Polish cancer patients receiving chemotherapy showed that cancer undoubtedly had a negative impact on all aspects of health, which was related to the disease process itself, the treatment used, and the duration of the disease [18]. Somatic symptoms accompany cancer patients at every stage of the disease and are associated with increased disability. The factors that significantly influence the occurrence of symptoms depend on the phase of the disease, the number of cycles of chemotherapy received, and the duration of the disease [19].

A follow-up study on HNC patients showed that depression increased with duration after cancer diagnosis and suggested that periodic screening of depression followed by psychological counseling for affected cancer patients should be offered during the course of treatment. The study also considered the negative impacts of late-stage diagnosis, substance abuse, lower education, socioeconomic background, and poor social-networking behavior as possible causes of depression, as compared to the positive impacts of good social support and emotional coping abilities [20]. Chemoradiation has been shown to have a maximal impact on depression scores, which deteriorate throughout the treatment due to side effects, such as pain, mucositis, breathing difficulties, and communication issues [21].

With respect to the coping domain, the present study showed that cancer patients from a rural background and those who were illiterate had higher scores. With respect to the positive focus coping subdomain, cancer patients who were male, from a rural background, and illiterate had higher scores. With respect to the diversion domain, rural and illiterate cancer patients had higher scores. With respect to the interpersonal scale domain, patients with a rural background, those belonging to a joint family, those >60 years of age, and illiterate patients had higher scores, and these differences were statistically significant (p<0.05).

Coping strategies are crucial mediators of the effects of stressful events in life. Coping with cancer refers to a set of attitudes and practices that one adopts to preserve health, well-being, and happiness, and to overcome the stresses of cancer. A similar study done on patients in India showed that disengagement (e.g., avoiding problems or giving up attempts to cope), which appeared to be a negative outcome, was higher in cancer patients [22]. The cancer patients predominantly used maladaptive strategies, such as self-blame and behavioral disengagement, to cope with cancer-related issues, with psychological distress being a very important factor in predicting the same. However, another Indian study showed that HNC patients adopted both emotion-oriented coping and problem-oriented coping during the course of illness, which was not affected by cancer site, treatment, age, education, or survival [23]. A study on HNC patients in Iran showed that age, gender, stage of cancer, mode of treatment, and disease duration had no statistically significant association with coping levels [24]. Although body image dissatisfaction in surgically treated HNC patients is an important factor in mental health, it is not typically addressed by the treating surgeon. This care gap highlights the importance of collaboration with mental health care physicians, for which institutions should consider new initiatives [25].

The present study showed that depression status had no impact on coping domains, and there was no significant correlation between depression scores and coping scores. A study done on HNC patients in Japan showed that there was a weak positive correlation between mental health status (e.g., depression) and coping scores in cancer patients [26]. HNC patients are significantly affected by facial disfigurement even after surgery and chemoradiation, which extremely hinders their mental health and coping abilities. Treating onco-surgeons must look out for coping strategies adopted by HNC patients, and those who do not cope adequately should be considered for more intensive intervention while remaining in the therapeutic environment of the hospital. The biggest hurdle that HNC patients face is the inability to cope with disfigurement and dysfunction at discharge, with the latter mostly remaining unrecognized [27]. 

Studies have established a relationship between intervention and the betterment of coping strategies and skills, along with mental health status, among HNC patients. Any psychosocial intervention can offer some help for HNC patients to deal with their condition, as they would most likely not have undergone any such emotional or problem-focused coping interventions. Such interventions before surgery or the initiation of chemotherapy, even just a few days prior, can significantly improve mental health and yield effective coping under these stressful conditions [28]. According to another systematic analysis, health interventions (e.g., educational, psychosocial, physical, and psychological symptom management), mindfulness, pharmacologic therapy, exercise, and telemedicine had a positive impact on the overall health of HNC patients [29]. These interventions can improve the quality of life of HNC patients.

The strength of this study is that we used a validated tool to evaluate depression and coping among cancer patients. The study was limited in that it was done in a single center. In addition, we included only HNC patients in this study. Each cancer has its own natural history and progression, which can deeply influence the mental health and coping strategy of cancer patients, making it extremely difficult to generalize the results among other cancers. There are many confounders that could not be captured which had direct or indirect relation in the causation of mental health issues and coping strategies. Quantification of symptoms because of cancer per se or cancer treatment like excessive salivation, eating problems, and emotional and social relationships can also play a huge role in altering the mental health status which was not taken into consideration. Mixed research which includes qualitative studies like focus group discussions and key informant interviews would be better to explore coping skills, which the present study did not explore.

## Conclusions

This is a cross-sectional study carried out for a period of three years among head and neck cancer patients to find out the prevalence of depression among HNC patients, assess coping strategies among HNC patients, and also to find out whether depression among HNC patients affects the coping strategies. Out of 188 cancer patients, 62.2% belonged to 31-60 years, 72.3% were females, 64.4% belonged to rural backgrounds, and 76.1% were illiterates. Thirty (16%) patients had one or other types of depression according to the Zung Depression Scale. HNC patients who had received intravenous chemotherapy had higher odds of having depression. Of various domains tested for coping among the same HNC patients, patients from a rural background, illiterates, and duration of illness of fewer than six months had better-coping scores suggesting a better adjustment to illness.

This study showed that depression was not associated with coping skills and strategies among HNC patients suggesting that tackling mental health issues should be adjunct with empowering adaptive, positive health engagement coping skills among HNC patients. Cancer treatment should be done with the intent of improving the survival of the diagnosed patient, which includes targeted mental health intervention. Mental health issues such as depression and anxiety are usually not considered by the treating oncologist. Such neglect by the treating team can adversely affect the overall health of the cancer patient and push them to maladaptive coping strategies, which could be ruinous for the patient as they may define their ability to face the reality of their life situation. Therefore, efforts must be made to develop more adaptive coping responses. As cancer is a traumatic stressor, special focus must be given to the treating doctor on mental health issues and coping with cancer during routine cancer treatment. Mental health programs at cancer-treating hospitals, such as counseling related to treatment and positive affirmations, need to be included for holistic care of the person experiencing such terminal illness.
